# Characterization of Two Potential Biocontrol *Bacillus* Strains Against Maize Stalk Rot

**DOI:** 10.3390/microorganisms13102255

**Published:** 2025-09-26

**Authors:** Zhiwei Feng, Mengyao Qin, Xiaobing Ma, Ruiyun Feng, Huifang Zhao, Yingchao Meng, Chunzhen Cheng

**Affiliations:** 1 Key Laboratory of Sustainable Dryland Agriculture of Shanxi Province, Shanxi Agricultural University, Taiyuan 030031, China; zhiweifeng@126.com (Z.F.); zhfsxty@163.com (H.Z.); 18835118130@163.com (Y.M.); 2 College of Horticulture, Shanxi Agricultural University, Taiyuan 030031, China; 15617782376@163.com (M.Q.); m2239743150@163.com (X.M.); 3 College of Agriculture, Shanxi Agricultural University, Taiyuan 030031, China; fengruiyun1970@163.com

**Keywords:** *Bacillus* spp., biological control agent, *Fusarium* diseases, plant-growth-promoting rhizobacteria, genome mining

## Abstract

Maize stalk rot (MSR) is one of the most devastating fungal diseases affecting maize worldwide. In recent years, biological control agents have emerged as an environmentally friendly and highly attractive strategy for managing MSR. In this study, two *Bacillus* strains—*B. subtilis* KP3P9 and *B. siamensis* K13C—were shown to effectively inhibit the growth of the MSR pathogen *Fusarium graminearum* in vitro. Pot experiments showed that inoculation with KP3P9 significantly increased plant height, stem width, above-ground part fresh weight, and total plant fresh weight, whereas K13C significantly improved the stem width and under-ground part fresh weight of maize seedlings (*p* < 0.05), demonstrating their plant-growth-promoting potential. Moreover, both strains markedly reduced the disease severity indices (DSIs) of maize seedlings, indicating that they can enhance maize resistance to the pathogen. Whole-genome sequencing using Oxford Nanopore (ONT) and Illumina technologies showed that the complete genomes of KP3P9 and K13C contained biosynthetic gene clusters involved in the biosynthesis of antimicrobial secondary metabolites, including fengycin, bacillibactin, subtilin, pulcherriminic acid, subtilosin A, bacilysin, and others. Moreover, both strains exhibited strong antagonistic activity against *F. solani* (the causal pathogen of apple replant disease), as well as *F*. *oxysporum* f. sp. *cubense* race 1 (*Foc*1) and tropical race 4 (*Foc*TR4) (pathogens responsible for banana wilt disease), with inhibition rates exceeding 70% in vitro. These results indicate that KP3P9 and K13C are promising biocontrol agents for MSR and other devastating *Fusarium* diseases.

## 1. Introduction

Maize (*Zea mays* L.), one of the three major food crops in the world, is important not only for human consumption but also for animal feeding and fuel production [1]. However, the worldwide maize industry is greatly threatened by maize stalk rot (MSR), resulting in significant reductions in yield, quality, and mechanical harvesting efficiency of maize [2,3]. In China, MSR causes ~10% annual yield loss on average, with losses surpassing 30% in some areas in certain years [4,5]. *Fusarium graminearum* and *F*. *verticillioides* are the dominant causal pathogens of MSR [6,7]. Other *Fusarium* spp., such as *F.* cf. *longipes* [8], *F. moniliforme*, *F. temperatum*, and *F. subglutinans* [9], have also been reported to be associated with this disease [10,11]. As *Fusarium* pathogens can produce mycotoxins that are harmful not only to livestock but also to humans, they also contribute to the mycotoxin contamination of maize [12]. MSR pathogens can infect maize throughout the entire growing season and can survive in soil for multiple years. Furthermore, the promotion of the ‘returning crop straw to the field’ policy has increased pathogen abundance in soil annually, intensifying the occurrence and severity of MSR [12].

In recent years, biocontrol agents have attracted great attention for their sustainable and environmentally friendly advantages [6,13]. Among the reported biocontrol agents, *Bacillus* strains are more prevalent and popular than other microorganisms, such as *Pseudomonas* and *Streptomyces* strains [14,15]. *B. siamensis* GL-02, isolated from rhizosphere soil affected by severe stalk rot, significantly inhibits the growth of *F. graminearum* [16]. *B. siamensis* M54, an intestinal bacterium isolated from *Allomyrina dichotoma*, promotes maize growth and reduces stalk lesions caused by *F. graminearum* infection in both seedlings and adult plants [1]. The soil-isolated *B. velezensis* B105-8 promotes maize growth and suppresses various MSR pathogens [17]. Additionally, other *Bacillus* strains, such as *B. velezensis* TSA32-1 [18] and *B. methylotrophicus* TA-1 [19], have also shown promise as effective biocontrol agents against MSR.

Although some progress has been made in mining biocontrol agents against MSR [16,17], they have not yet been widely applied in the field. Therefore, continued in-depth exploration of biocontrol agents for this disease is of great significance. In the study, we performed bacteria isolation experiments from apple rhizosphere soil and evaluated their antagonistic activity against the dominate MSR pathogen *F. graminearum*. Two strains, KP3P9 and K13C, demonstrated strong in vitro anti-fungal activity against *F. graminearum.* Molecular characterization identified KP3P9 as *B. subtilis* and K13C as *B. siamensis.* To explore their possible functional mechanisms, we assessed their physiological and biochemical characteristics, investigated the influences of their inoculation on maize seedling MSR resistance, and assembled their genomes. The results obtained in this study will be helpful to elucidate the antagonistic mechanism of *Bacillus* strains against MSR pathogens and can provide a basis for the future applications of KP3P9 and K13C in controlling MSR and other *Fusarium* diseases.

## 2. Materials and Methods

### 2.1. Plant and Fungal Materials

The seeds of the *Zea mays* cv. ‘Xinruipu 826’ used in this study were provided by the College of Agriculture, Shanxi Agricultural University. This cultivar was bred by Shanxi Agricultural University and is one of the leading maize cultivars in Shanxi Province, China. Its cultivation region includes the provinces of Heilongjiang, Liaoning, Jilin, Hebei, and Shanxi, the autonomous region of Inner Mongolia, and the municipality of Tianjin, covering approximately 190,000 ha. The *F. graminearum*, *F. solani*, *F. oxysporum* f. sp. *cubense* race 1 (*Foc*1), and *F. oxysporum* f. sp. *cubense* tropical race 4 (*Foc*TR4) strains used in this study were previously isolated and purified by our lab, and the pathogenicity of these strains has been verified.

### 2.2. Isolation and In Vitro Antagonistic Activity of Bacterial Strains Against F. graminearum

Apple roots were gently removed from the soil, and loosely adhering soil was shaken off. Rhizosphere soil tightly attached to the root surface was then gently brushed off. Using a stepwise dilution-plating method, we isolated and purified bacteria from apple rhizosphere soil on lysogeny broth (LB) agar media (Ararat (Canton) Biotechnology Co., Ltd., Guangzhou, China). Subsequently, the isolated rhizosphere bacteria were subjected to antagonistic activity analysis against the MSR pathogen *F. graminearum* using dual-culture assays on potato dextrose agar (PDA)(Ararat (Canton) Biotechnology Co., Ltd., Guangzhou, China). Briefly, bacterial suspension was streaked in two parallel lines 2 cm away from the center of a 9 cm PDA plate on opposite sides. One day later, a 5 cm mycelial plug of *F. graminearum* was placed at the center of the PDA plate. After incubation at 28 °C in the dark for 7 d, two bacterial strains (KP3P9 and K13C) that can suppress fungal growth were selected for further analysis. To quantify their in vitro inhibition effects, the colony diameter of *F. graminearum* was measured daily. Plates inoculated with *F*. *graminearum* alone were used as controls. The inhibition rate was calculated using the following formula: Inhibition rate (%) = [(colony diameter of the control *F. graminearum* − colony diameter of KP3P9/K13C treated *F. graminearum*)/colony diameter of the control *F. graminearum*] × 100. Each strain was tested in six replicates.

### 2.3. Plant-Growth-Promoting Assays of KP3P9 and K13C on Maize Seedlings

Using the seed-soaking method, we evaluated the effects of KP3P9 and K13C on the growth of maize (cv. ‘Xinruipu 826’) seedlings. Single colonies of KP3P9 and K13C were separately inoculated into sterile LB and incubated at 37 °C with shaking at 200 rpm in a constant temperature shaking incubator (Shanghai Yiheng Scientific Instrument Co. Ltd., Shanghai, China) for 24 h. Cultures were adjusted to OD_600_ ≈ 1.0 using sterile LB and used as bacterial inoculum. ‘Xinruipu 826’ seeds were allocated to three groups (control (CK), KP3P9, and K13C; 80 seeds each). CK seeds were soaked in sterile LB for 1 h, whereas seeds of the KP3P9 and K13C groups were soaked for 1 h in corresponding bacterial inoculum. The seeds were then sown in plug trays (Suzhou Huanmei Horticulture Technilogy Co. Ltd., Suzhou, China) (8 cm diameter, 10 cm height) filled with substrates (with an organic matter content of ≥35% and a neutral pH) and grown in a 25 °C greenhouse under natural light conditions. The substrates used for maize cultivation were purchased from Shen County Luyuan Seedling Matrix Co., Ltd. (Liaocheng, China). Seedlings were watered with 1/2 Hoagland modified nutrient salts solution (Shanghai Zeye Biotechnology Co., Ltd., Shanghai, China) every two days.

Two weeks after sowing, plant height, stem diameter, above-ground part fresh weight, under-ground part fresh weight, and total plant fresh weight were measured. The plant height was measured using a ruler. The stem diameter was measured using a vernier caliper (Shanghai Measuring Tool and Cutting Tool Factory Co., Ltd., Shanghai, China). The fresh weights of above-ground and under-ground parts and the whole seedling were measured with an electronic balance (HAT-A+100, Huazhi (Fujian) Electronics Technology Co., Ltd., Putian, China). Root architecture was analyzed using a root phenotypic analysis system (ScanMaker i800 Plus GXY-A, Hangzhou, China) to obtain total root length, average root diameter, root volume, root surface area, root projected area, and root tip number. Each parameter was assessed using at least six maize seedlings from each group.

### 2.4. The Biocontrol Activity Analysis of KP3P9 and K13C Against MSR in Maize Seedlings

The effects of KP3P9 and K13C on the MSR resistance of ‘Xinruipu 826’ maize seedlings were also assessed. Seeds were soaked in either the KP3P9 or K13C bacterial suspension for 1 h, sown in plug trays (8 cm diameter, 10 cm height) containing substrates that had been fully drenched with the corresponding bacterial suspension (OD_600_ ≈ 1.0), and then cultivated in a 25 °C greenhouse under natural light conditions. Two weeks after sowing, each maize seedling was watered twice with *F. graminearum* conidial suspension (1 × 10^5^ conidia mL^−1^, 4 mL per seedling). Two weeks post pathogen inoculation, the disease severity of each seedling was evaluated using the disease severity index (DSI) analysis. The DSI of maize seedlings from different groups were calculated using the following formula: DSI (%) = ∑(scale value × number of maize seedlings with each scale value)/(total number of maize seedlings) × 100 [20]. For each group, at least 24 maize seedlings were used.

### 2.5. Molecular Characterization of KP3P9 and K13C

The *16S rRNA* genes of KP3P9 and K13C were amplified using the universal primer pair 27F/1492R. PCR was performed in a 25 µL reaction mixture containing 12.5 µL of 2× rTaq Mix, 1 µL of each primer (10 µM), 1 µL of genomic DNA template, and 9.5 µL of ddH_2_O. The amplification program was as follows: initial denaturation at 95 °C for 5 min; 32 cycles of 95 °C for 30 s, 51 °C for 30 s, and 72 °C for 1 min; and a final extension at 72 °C for 10 min. PCR products were purified and sequenced by Shanghai Sangon Biotech Co., Ltd. (Shanghai, China). The *16S rRNA* gene sequences were deposited in GenBank under the accession IDs of PV759774 for KP3P9 and PV759773 for K13C. Then, the *16S rRNA* sequences of KP3P9 and K13C were queried against the NCBI nucleotide database using Blastn. Their homologous *16S rRNA* sequences were downloaded and used to construct a neighbor-joining tree using MEGA11 with default parameters.

### 2.6. Physiological and Biochemical Characterization of B. subtilis KP3P9 and B. siamensis K13C

According to the methods described by Dong and Cai [21], the amylase activity, urease activity, catalase activity, nitrate reduction ability, and H_2_S-producing ability of KP3P9 and K13C strains were examined. Moreover, the methyl red test and the Voges–Proskauer (VP) test were also performed on them [21].

### 2.7. Phosphate-Solubilizing and Potassium Release Capacity Analysis of KP3P9 and K13C

KP3P9 and K13C cultures were streaked onto LB agar plates and incubated at 37 °C in the dark for 16 h. Agar plugs (5 mm, fully covered by bacteria) were transferred to Pikovaskaia’s inorganic phosphorus medium (Ararat (Canton) Biotechnology Co., Ltd., Guangzhou, China), Monia’s organic phosphorus medium (Ararat (Canton) Biotechnology Co., Ltd., Guangzhou, China) and Aleksandrov’s medium (Ararat (Canton) Biotechnology Co., Ltd., Guangzhou, China) to determine the inorganic phosphate dissolution, organic phosphate dissolution, and potassium release activities of each of the two *Bacillus* strains [22,23].

### 2.8. Indole-3-Acetic Acid (IAA)-Producing Capacity Analysis of KP3P9 and K13C

Using the Salkowski colorimetric method [24], the IAA-producing capacity of KP3P9 and K13C was determined. Briefly, a bacterial colony was inoculated into LB medium (containing 200 mg/L l-tryptophan) and incubated at 37 °C with shaking at 180 r/min for 2 d. The culture was then centrifuged at 8000 r/min for 5 min to obtain the supernatant. Two milliliters of the supernatant were mixed with an equal volume of Salkowski reagent to initiate the color reaction, with LB medium (containing 200 mg/L l-tryptophan) and 50 mg/L IAA solution used as the negative and positive control, respectively.

### 2.9. Determinations of Cellulase, Protease, β-1,3-Glucanase and Siderophore-Producing Activities

KP3P9 and K13C bacterial plugs were inoculated onto Congo red cellulose medium (Ararat (Canton) Biotechnology Co., Ltd., Guangzhou, China), skim milk medium (Ararat (Canton) Biotechnology Co., Ltd., Guangzhou, China), and β-1,3-glucanase detection medium (Ararat (Canton) Biotechnology Co., Ltd., Guangzhou, China) to determine the ability of these strains to secrete cellulase [25], protease [26], and β-1,3-glucanase [27]. Siderophore-producing activity was evaluated on chrome azurol S (CAS) agar (Ararat (Canton) Biotechnology Co., Ltd., Guangzhou, China) [28].

### 2.10. Genome Sequencing and Assembly

Using the Ezup column bacteria genomic DNA purification kit (B518255-010, Sangon Biotech, Shanghai, China), high-quality KP3P9 and K13C genomic DNA were extracted. High-molecular-weight DNA fragments were size-selected using a BluePippin automated nucleic-acid collection system. Libraries were prepared with the SQK-LSK109 ligation sequencing kit (Oxford Nanopore Technologies, Oxford, UK)and sequenced on a PromethION 48 platform by Biomarker Technologies Co., Ltd. (Beijing, China). After removing adapter sequences, low-quality bases, and short reads (<2000 bp), the final clean reads were obtained. Initial assembly was conducted with Canu v1.5, followed by mapping of Illumina short reads to the draft assembly and polishing with Pilon v1.19. Contigs were aligned against the NCBI nucleotide (nt) database to assign chromosomal origin. Protein-coding genes, repetitive elements, tRNAs, and rRNAs/other ncRNAs were predicted using Prodigal v2.6.3, RepeatMasker v4.0.5, tRNAscan-SE v2.0, and Infernal v1.1.3 (Rfam), respectively. CRISPR (clustered regularly interspaced short palindromic repeats) arrays were identified using CRT v1.2, genomic islands using IslandPath-DIMOB v2.0, prophages using PhiSpy v2.3, and biosynthetic gene clusters (BGCs) using antiSMASH v5.0.0.

### 2.11. Antagonistic Activity of the Two Bacillus Strains Against Other Fusarium Pathogens

Dual-culture assays were performed to evaluate the antagonistic potential of strains KP3P9 and K13C against *Fusarium solani* (a pathogen associated with apple replant disease) and the banana wilt pathogens *F. oxysporum* f. sp. *cubense* race 1 (*Foc*1) and tropical race 4 (*Foc*TR4) [4]. A 5 mm mycelial plug of each *Fusarium* isolate was placed at the center of a PDA plate that had been streaked with two parallel lines of KP3P9 or K13C suspension for 1 d. Plates were incubated at 28 °C in the dark for 7 d. Colony diameters were measured every day, and their inhibition rates against the three *Fusarium* isolates were calculated as described in Section 2.1.

### 2.12. Statistical Analysis

All data were presented as mean ± standard deviation (SD) of at least three biological replicates. Statistical analysis was performed using SPSS 27.0, with one-way ANOVA followed by Duncan’s multiple range test at a significance level of *p* < 0.05.

## 3. Results

### 3.1. Morphological, Physiological and Biochemical Characteristics of KP3P9 and K13C

**Figure 1:**
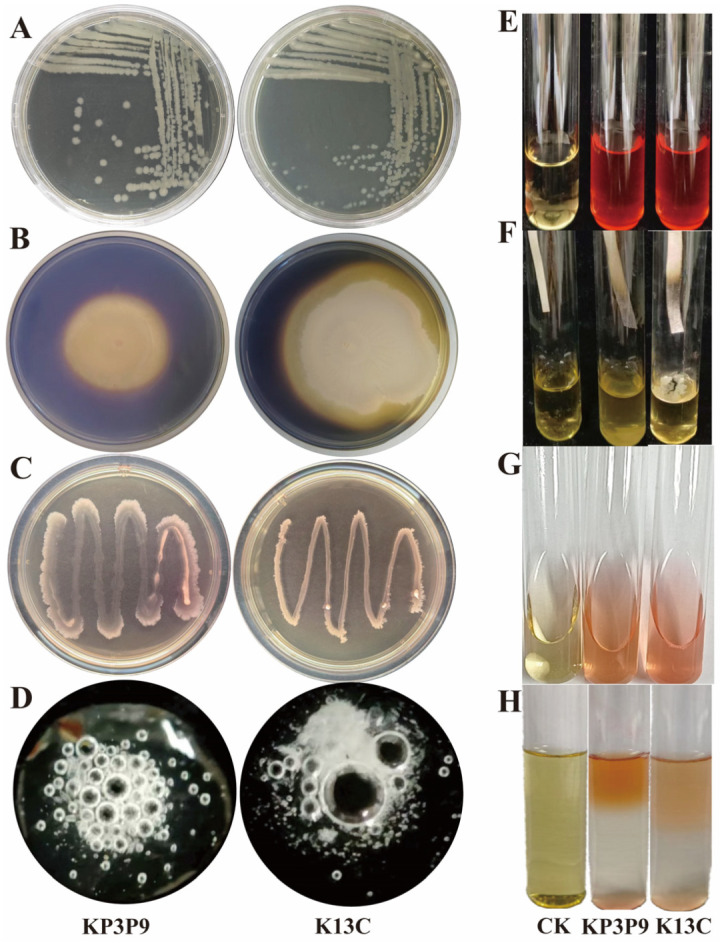
Colony morphology and physiological–biochemical characteristics of KP3P9 and K13C. (**A**) Colony morphology characteristics of KP3P9 (left) and K13C (right) on LB medium. (**B**–**H**) Detection results of amylase ability (**B**), urease activity (**C**), catalase activity (**D**), nitrate reduction ability (**E**), H_2_S-producing ability (**F**), V-P test (**G**) and methyl red test (**H**). In (**B**): A transparent ring around the colony indicates a positive result; otherwise, the result is negative. In (**C**) a change in the culture medium from yellow to red indicates a positive result; if the medium remains yellow, the result is negative. In (**D**) a positive result is indicated by bubble formation; absence of bubbles signifies a negative result. In (**E**,**G**,**H**) a red color change in the solution indicates a positive result. In (**F**) a positive result is indicated by the filter paper strip turning black.

Bacteria isolated from the apple rhizosphere soil were screened for antagonism against *F. graminearum* by dual-culture assay. Results showed that two bacterial strains, KP3P9 and K13C, strongly inhibited the growth of the pathogen causing MSR. On LB medium, colonies of both KP3P9 and K13C were light-yellow, moist, and smooth (Figure 1). The physiological and biochemical characteristics of the two strains were studied (Figure 1B–H). Results showed that both KP3P9 and K13C had the ability to produce amylase (Figure S1B), catalase (Figure 1D), and H_2_S (Figure 1F), and reduced nitrate (Figure 1E), but neither exhibited urease activity (Figure 1C). Moreover, both strains exhibited positive reactions in the V-P test (Figure 1G) and methyl red test (Figure 1H).

### 3.2. Antagonistic Activity of KP3P9 and K13C Against F. graminearum

**Figure 2:**
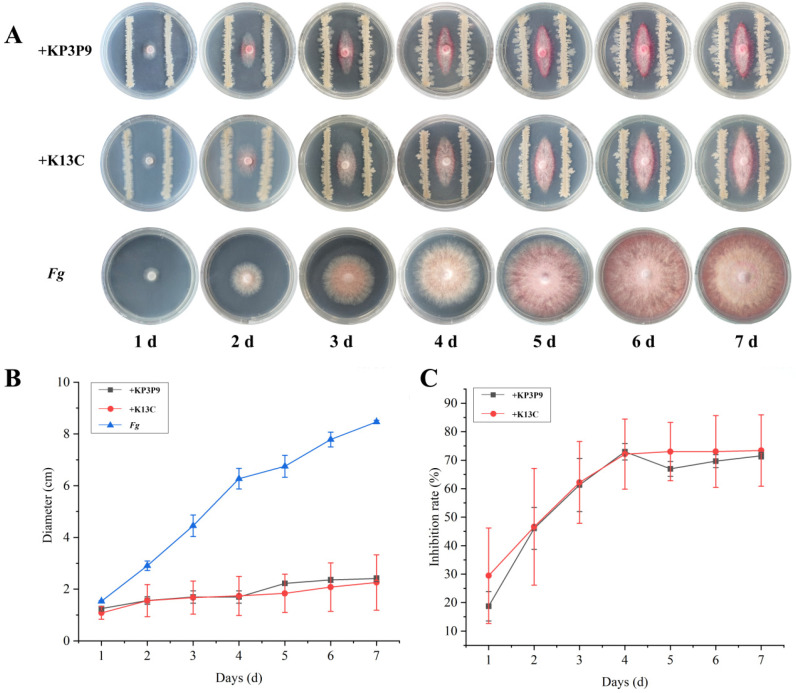
Antagonistic activity of KP3P9 and K13C against *F. graminearum* on PDA medium. (**A**) Dual-culture assay results for KP3P9 /K13C and *F. graminearum* on PDA medium. (**B**) Colony diameters for *F. graminearum* cultured alone or co-cultured with KP3P9/K13C on PDA medium. (**C**) Inhibitory effect of KP3P9/K13C on the growth of *F. graminearum*. *Fg*: *Fusarium graminearum*.

We further systematically examined the inhibitory effects of *KP3P9* and *K13C* on *F. graminearum* using dual-culture assays. Results showed that both of the strains markedly suppressed *F. graminearum* mycelial growth on PDA media (*p* < 0.05) (Figure 2A). After seven days of confrontation, the pathogen colonies were sparse and their radial expansion was significantly reduced (Figure 2B), yielding inhibition rates of 71.58% for KP3P9, and 73.4% for K13C (Figure 2C).

### 3.3. Effects of KP3P9 and K13C on the Growth of Maize Seedlings

**Figure 3:**
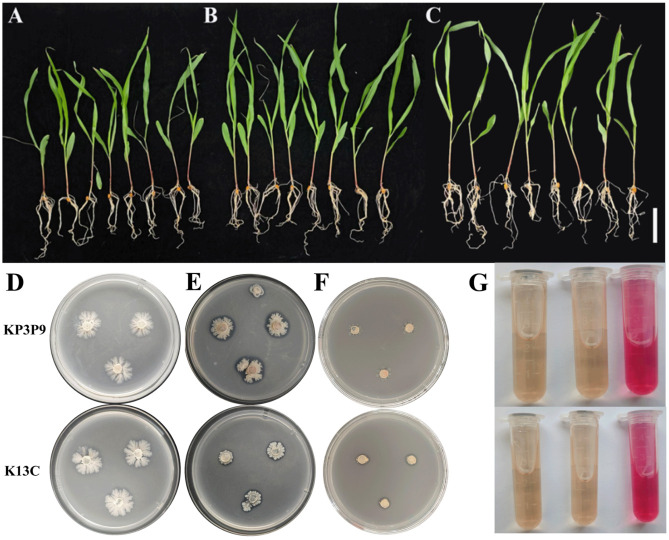
Effects of KP3P9 and K13C inoculation on the growth of maize seedlings. (**A**–**C**) Typical phenotypes for non-inoculated (**A**), KP3P9-inoculated (**B**) and K13C-inoculated (**C**) maize seedlings. Bar = 10 cm. (**D**–**F**) Assay results for organic-phosphate solubilization (**D**), inorganic-phosphate solubilization (**E**), potassium release (**F**), and IAA production (**G**) tests. In (**D**–**F**) a transparent ring around the colony indicates a positive result; otherwise, the result is negative. (**G**) Left, negative control; middle, treatment; right, positive control.

The influences of KP3P9 and K13C inoculation on the growth of maize seedlings were studied (Figure 3A–C, Table 1). KP3P9 inoculation significantly increased the plant height, stem width, above-ground part fresh weight, and total plant fresh weight of maize seedlings (*p* < 0.05): 1.23-, 1.20-, 1.55- and 1.31-fold compared to the non-inoculated controls (CK), respectively. Although the under-ground part fresh weight of KP3P9-treated maize seedlings was slightly lower than CK, no significant difference was identified between them. K13C inoculation significantly improved the stem width and under-ground part fresh weight of maize seedlings (*p* < 0.05), accounting for 1.21- and 1.15-fold increases compared to CK, respectively. Moreover, although no significant difference was identified, the plant height, above-ground part fresh weight, and total plant fresh weight of K13C-inoculated maize seedlings increased by approximately 1.07-, 1.21- and 1.19-fold compared to CK, respectively.

The influences of KP3P9 and K13C inoculation on the root architecture-related parameters were further studied. Although no significant difference was identified among the three groups, the average total root length, root diameter, root volume, root surface area, root projected area, and root tip number of the KP3P9 and K13C groups were all higher than those of the CK group (Table 1). This finding indicated that the inoculation of the two strains can improve the root development of maize seedlings.

To elucidate the mechanism underlying the root and plant growth-promoting effects of KP3P9 and K13C, we assessed their abilities of solubilizing organic and inorganic phosphates (Figure 3D,E), releasing potassium (Figure 3F) and producing IAA (Figure 3G). Results showed that K13C exhibited both organic and inorganic phosphate-solubilizing, and potassium-releasing capacities (Figure 3D,E), whereas KP3P9 only demonstrated inorganic phosphate-solubilizing activity (Figure 3E). Moreover, neither of them was capable of producing IAA (Figure 3G).

### 3.4. Influences of KP3P9 and K13C on the MSR Resistance of Maize Seedlings

**Figure 4:**
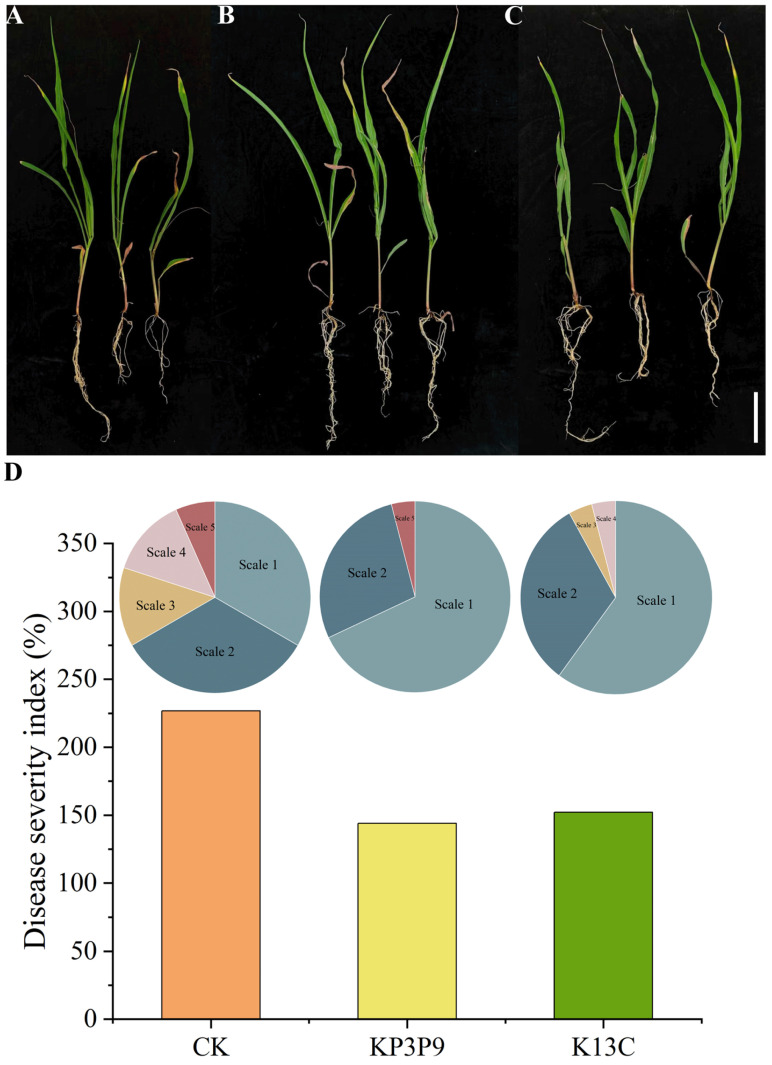
Biocontrol efficacy of KP3P9 and K13C against MSR. (**A**–**C**) Representative phenotypes of maize seedlings from CK, KP3P9, and K13C groups at two weeks post *F. graminearum* inoculation. Bar = 10 cm. (**D**) Disease severity index (DSI) for maize seedlings from CK, KP3P9, and K13C groups. DSI was calculated using a 5-point classification scale (from Scale 1 (the most resistant) to Scale 5 (the most susceptible)).

We evaluated the biocontrol efficacy of KP3P9 and K13C against MSR in ‘Xinruipu 826’ seedlings. At two weeks after *F. graminearum* inoculation, maize seedlings of the KP3P9 and K13C groups exhibited markedly milder disease symptoms than those of the CK group (Figure 4A–C). The disease severity of individual seedlings was scored on a 5-point scale [20]. Compared with CK, both KP3P9 and K13C increased the proportion of seedlings rated as Scale 1 (most resistant) and decreased the proportion rated as Scales 3–5 (Figure 4D). Consequently, disease severity indices (DSIs) were 226.67% for CK, 144% for KP3P9, and 152% for K13C (Figure 4D). These findings indicated that KP3P9 and K13C enhanced the MSR resistance of maize seedlings.

### 3.5. Molecular Identification of KP3P9 and K13C

**Figure 5:**
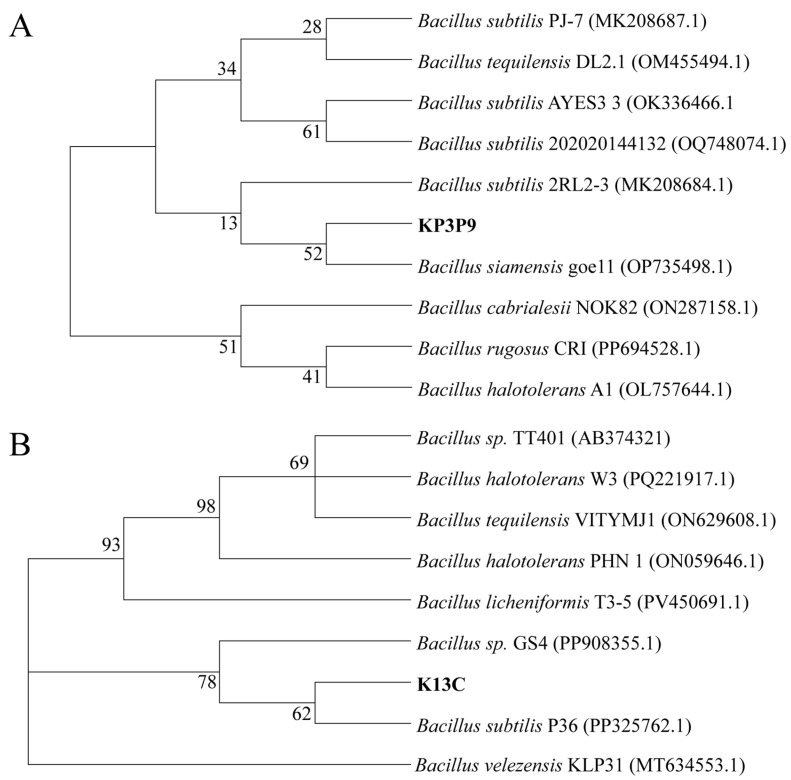
Phylogenetic analysis results using *16S rRNA* sequences of KP3P9 (**A**), K13C (**B**), and other *Bacillus* strains.

For the molecular identification of the two bacteria, their *16S rRNA* genes were amplified, sequenced, and subjected to phylogenetic analysis. Phylogenetic analysis revealed that KP3P9 clustered with *Bacillus subtilis* P36 (PP325762.1) and K13C with *Bacillus siamensis* geo11 (OP735498.1) (Figure 5), leading to their designation as *B. subtilis* KP3P9 and B. *siamensis* K13C, respectively. Their *16S rRNA* sequences have been deposited in GenBank under the accession numbers PV759774 (KP3P9) and PV759773 (K13C).

### 3.6. Genome Assembly and Annotation Results of KP3P9 and K13C, and Prediction Results of Biosynthetic Gene Clusters

To further elucidate the functional mechanisms of KP3P9 and K13C, their whole-genome sequences were obtained, assembled, and annotated (Supplemental Figure S1). The chromosome genome of KP3P9 is 4,279,804 bp in length, with a G + C content of 43.57%. A total of 4540 protein coding genes, 30 rRNA, and 97 tRNA genes were predicted. The KP3P9 genome also contains one 5553 bp repetitive sequence (0.13% of the chromosome), four prophage sequences, six gene islands, and four CRISPRs (Table 2). The genome of strain K13C is 4,066,559 bp, with a G + C content of 43.8%. Its genome comprises 3990 protein coding genes, 27 rRNA genes, and 86 tRNA genes, along with one 4005 bp repetitive sequence (0.10% of the total length), four prophage sequences, six gene islands, and five CRISPRs (Table 2). The genome sequences of KP3P9 and K13C have been deposited in the GenBank database under accession numbers JBQIBB000000000 and JBQIBC000000000, respectively.

Antimicrobial activity of microorganisms is largely attributed to the secondary metabolites they produce. Biosynthetic gene clusters (BGCs) prediction analysis showed that both K13C and KP3P9 contain 14 BGCs (Table 3). In KP3P9, eight clusters are identical to known BGCs involved in the biosynthesis of sporulation killing factor: bacillaene, fengycin, sublancin 168, bacillibactin, pulcherriminic acid, subtilosin A, and bacilysin. Three additional clusters (3, 6 and 13) are of unknown function. In K13C, seven clusters are identical to known BGCs for the production of surfactin, fengycin, bacillibactin, subtilin, pulcherriminic acid, subtilosin A, and bacilysin, while four clusters (2, 3, 6 and 13) are uncharacterized. Furthermore, both strains contain three BGCs with varying degrees of similarity to known clusters, 82% similarity to the surfactin BGC, 16% to the 1-carbapen-2-em-3-carboxylic acid BGC, and 10% to the thailanstatin A BGC.

### 3.7. Potential Antimicrobial Substrances Detection Results

**Figure 6:**
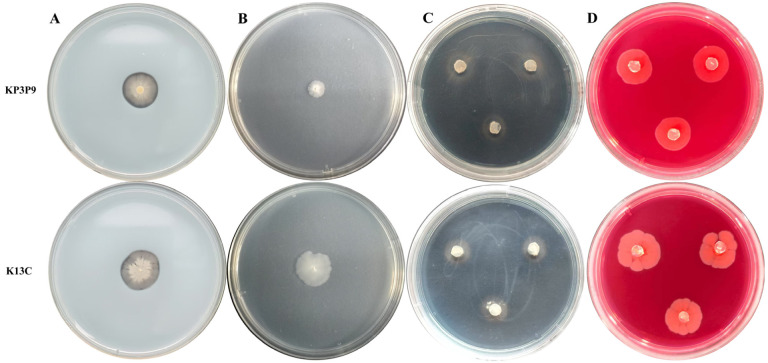
Protease (**A**), β-1,3-glucanase (**B**), siderophore-producing (**C**), and cellulose (**D**) activities analysis results for KP3P9 and K13C. In (**A**,**B**,**D**) presence of a transparent ring around the colony indicates a positive result; absence indicates a negative result. In (**C**) an orange-yellow halo indicates positive siderophore secretion.

We further investigated the abilities of strains KP3P9 and K13C to produce protease, β-1,3-glucanase, siderophores, and cellulase. Results showed that both strains exhibited protease- and siderophore-producing activities, whereas neither showed cellulase or β-1,3-glucanase activity (Figure 6).

### 3.8. The Antagonistic Ability of KP3P9 and K13C Against Other Fusarium Species

**Figure 7:**
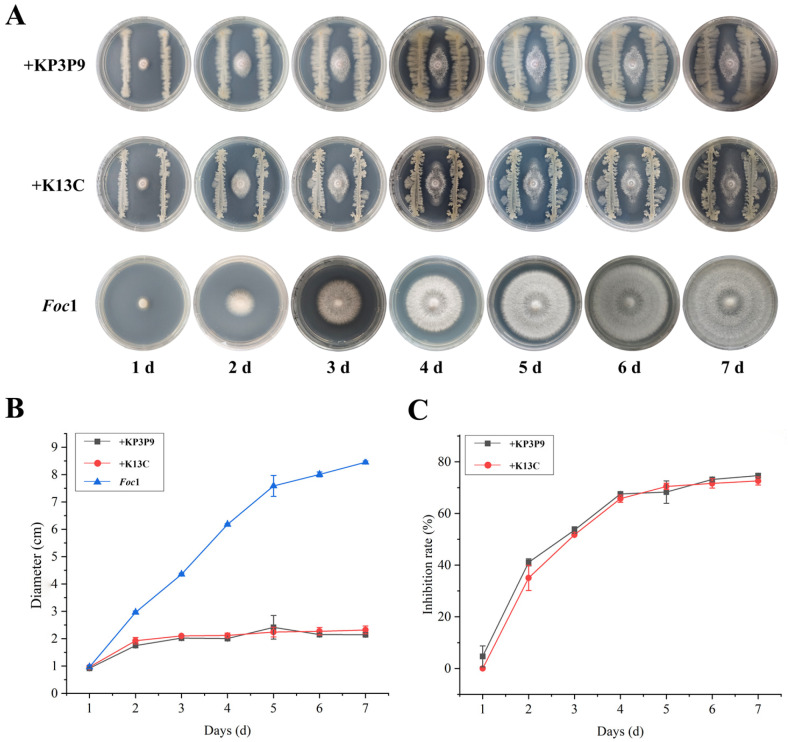
Antagonistic activity of KP3P9 and K13C against *F*. *oxysporum* f. sp. *cubense* race 1 (*Foc*1). (**A**) Dual-culture assay results for KP3P9/K13C and *Foc*1 on PDA medium. (**B**) Colony diameters for *Foc*1 cultured alone or co-cultured with KP3P9/K13C on PDA medium. (**C**) Inhibitory effect of KP3P9/K13C on the growth of *Foc*1.

**Figure 8:**
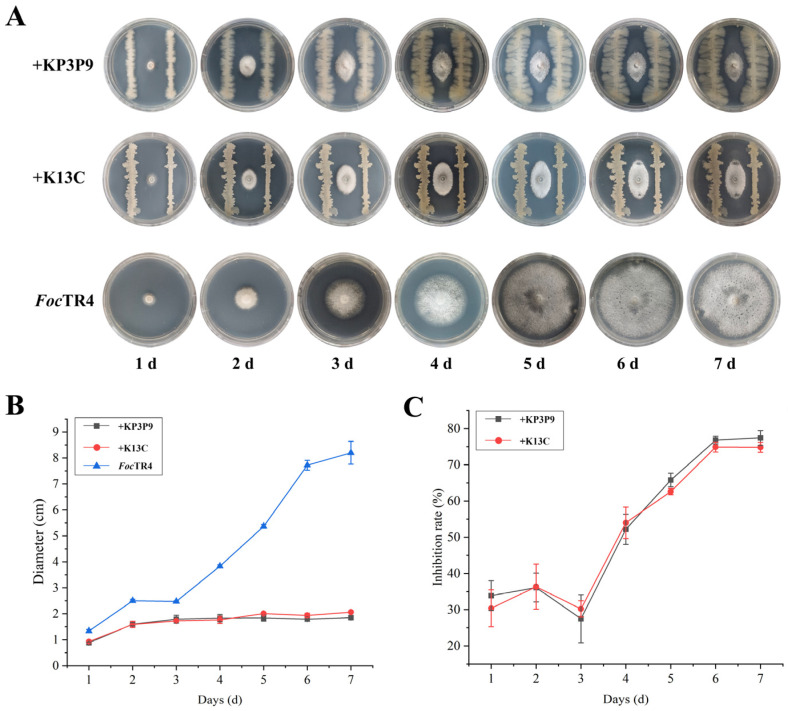
Antagonistic activity of KP3P9 and K13C against *F*. *oxysporum* f. sp. *cubense* tropical race 4 (*Foc*TR4). (**A**) Dual-culture assay results for KP3P9 /K13C and *Foc*TR4 on PDA medium. (**B**) Colony diameters for *Foc*TR4 cultured alone or co-cultured with KP3P9/K13C on PDA medium. (**C**) Inhibitory effect of KP3P9/K13C on the growth of *Foc*TR4.

**Figure 9:**
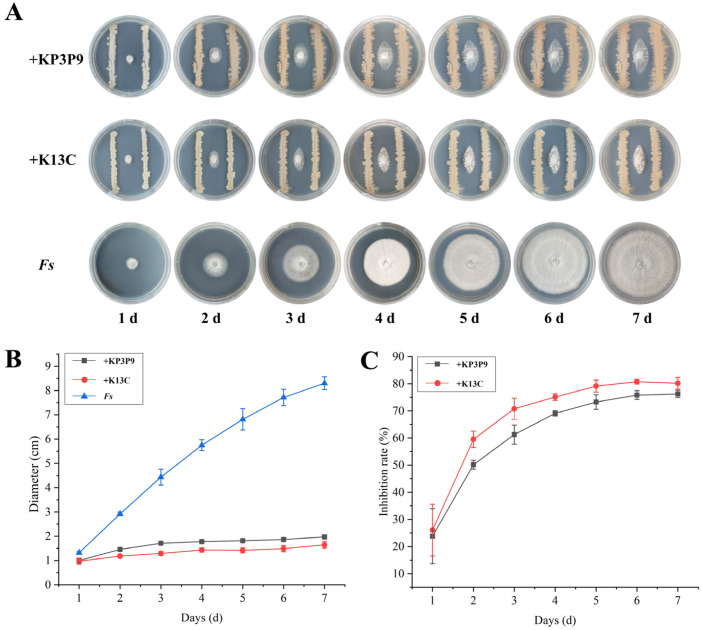
Antagonistic activity of KP3P9 and K13C against *F. solani* (*Fs*). (**A**) Dual-culture assay results for KP3P9 /K13C and *Fs* on PDA medium. (**B**) Colony diameters for *Fs* cultured alone or co-cultured with KP3P9/K13C on PDA medium. (**C**) Inhibitory effect of KP3P9/K13C on the growth of *Fs*.

Both *Bacillus* strains KP3P9 and K13C contain numerous BGCs that are predicted to be associated with the biosynthesis of anti-fungal metabolites, suggesting broad-spectrum activity against fungal pathogens. Using dual-culture assays, we evaluated their antagonistic effects on the banana wilt pathogens *Fusarium oxysporum* f. sp. *cubense* race 1 (*Foc*1) and tropical race 4 (*Foc*TR4), as well as on *Fusarium solani* (*Fs*), the causal agent of apple replant disease. After seven days of co-culture, KP3P9 and K13C significantly suppressed the radial growth of *Foc*1 by 74.5% and 72.6% (Figure 7), of *Foc*TR4 by 77.4% and 74.8% (Figure 8), and of *Fs* by 76.3% and 80.2% (Figure 9), respectively. These results demonstrate that KP3P9 and K13C possess broad-spectrum anti-fungal activity against *Fusarium* spp.

## 4. Discussion

In this study, we found that the inoculations of the two *Bacillus* strains (*B. siamensis* KP3P9 and *B. subtilis* K13C) increased the plant height and biomass of maize seedlings, confirming their plant-growth-promoting (PGP) ability. Both KP3P9 and K13C possess siderophore-producing, inorganic phosphate-solubilizing, nitrate-reducing, and potassium-releasing capacities, suggesting that their PGP effects are mediated by improved soil fertility and enhanced root nutrient uptake [29,30,31,32]. Moreover, K13C also solubilizes organic phosphate, indicating that the two strains employ partially distinct mechanisms to promote maize growth.

Our study found that, in vitro, KP3P9 and K13C inhibited *F. graminearum* growth by 71.6% and 73.4%, respectively. Moreover, their inoculation reduced the DSI of maize seedling resistance to MSR. These results demonstrate that KP3P9 and K13C are promising biocontrol agents against MSR. *Bacillus* spp. have been successfully applied as biocontrol agents against several crop diseases [33,34,35]. Both *B. siamensis* and *B. subtilis* have been shown to be effective biocontrol agents against fungal pathogens [36,37]. *B. siamensis* GL-02 and M54 effectively inhibit *F. graminearum* and reduce stalk-rot lesions in maize [1,16]. *B. subtilis* SL-44 enhances root defense-related enzyme activities, increases beneficial microbial abundance in soil, and directly inhibits *Rhizoctonia solani* (Rs), thereby improving resistance of *Brassica chinensis* L. to Rs [38]. *B. subtilis* JNF2, isolated from an area with high incidence of cucumber *Fusarium* wilt, significantly suppresses *F. oxysporum* f. sp. *cucumerinum* [39]. *B. subtilis* IBFCBF-4 exhibits high biocontrol potential against *Fusarium* wilt of watermelon caused by *F. oxysporum* f. sp. *niveum* [40]. *B. subtilis* SF1 effectively controls potato wilt caused by *F. foetens*, achieving 52.5 ± 2.6% mycelial growth inhibition in vitro and reducing disease incidence by 45.6% under field conditions [41]. *B. subtilis* BS06 is a promising biocontrol agent against *F. oxysporum*, the causal agent of soybean root rot [42].

Proteases play important roles in inhibiting pathogen growth and infection, as well as in enhancing the disease resistance of plants [43]. Siderophores have been identified as anti-fungal agents and can induce systemic resistance of plants [44]. Here, we found that both KP3P9 and K13C exhibited protease- and siderophore-producing activities, which might be closely associated with their anti-fungal activities.

By assessing their antagonistic effects on the fungi that cause banana wilt and apple replant disease, we found that both KP3P9 and K13C exhibited strong antagonism against these pathogens. Genome sequencing yielded assemblies of 4,440,737 bp (KP3P9) and 4,279,804 bp (K13C), each containing 14 BGCs. The numbers of BGCs in KP3P9 and K13C differ from those of other *B. subtilis* and *B. siamensis* strains. For example, the genomes of *B. subtilis* TY-1 [45], *B. subtilis* N4 [46], *B. siamensis* YB-1631 [47], and *B. siamensis* SCSIO 0574 [48] contain 10, 11, 10, and 19 BGCs, respectively. These findings suggest that the different antimicrobial activities among *Bacillus* spp. are associated with the types and abundance of BGCs in their genomes. Both KP3P9 and K13C genomes contain BGCs that are involved in the biosynthesis of fengycin, bacillibactin, subtilin, pulcherriminic acid, subtilosin A, and bacilysin, which have been repeatedly implicated in pathogen suppression. The fengycin isolated from *B. amyloliquefaciens* FZB42 significantly suppresses the growth of *F. graminearue* and reduces disease damage. Moreover, fengycin can also reduce the mycotoxin production in infected grains [49]. Bacillibactin [50], subtilin (and subtilin-like) [51], pulcherriminic acid [52], subtilosin A [53], and bacilysin [54] have all been reported to have inhibitory effects on microbial pathogens. The presence of BGCs encoding these antibiotic metabolites suggests that they are closely related to their anti-fungal abilities. Furthermore, KP3P9 additionally carries the sporulation killing factor and the *B. subtilis* 168 cluster, whereas K13C has the surfactin cluster—both of which also contribute to pathogen inhibition [55,56]. Collectively, our study indicated that the anti-fungal activities of the two *Bacillus* strains are closely associated with the BGCs encoding antimicrobial metabolites.

## 5. Conclusions

In this study, two *Bacillus* species (*B. subtilis* K13C and *B. siamensis* KP3P9) isolated from apple rhizosphere soil were found to promote maize seedling growth, suppress the MSR pathogen *F. graminearum* in vitro, and reduce the DSI of *F. graminearum*-inoculated seedlings in pot experiments. Both strains exhibited siderophore production, nitrate reduction, inorganic phosphate solubilization, and protease activity—traits linked to PGP and anti-fungal effects. In the future, field experiments should be conducted to verify their PGP and anti-fungal effects on maize. Genome mining revealed BGCs for several anti-fungal metabolites, likely contributing to their broad-spectrum antagonism against *Fusarium* pathogens. To further identify the key genes and metabolites driving their anti-fungal activity, transcriptomic and metabolomic analyses of the two bacteria are warranted. Moreover, to clarify their functional mechanisms, their influences on the expression of growth- and defense-related genes and on the activities of related enzymes should also be studied.

## Figures and Tables

**Table 1:** The influences of KP3P9 and K13C on the growth of maize seedlings. Different letters in the same line indicate significant difference among samples from CK, KP3P9 and K13C groups (*p* < 0.05).

Parameters	CK	KP3P9	K13C
Plant height (cm)	37.92 ± 3.04 b	46.50 ± 2.32 a	40.40 ± 4.13 b
Stem width (mm)	2.57 ± 0.32 b	3.09 ± 0.37 a	3.12 ± 0.22 a
Above-ground part fresh weight (g)	1.67 ± 0.35 b	2.59 ± 0.32 a	2.02 ± 0.29 b
Under-ground part fresh weight (g)	1.18 ± 0.15 b	1.14 ± 0.03 b	1.36 ± 0.04 a
Plant fresh weight (g)	2.85 ± 0.36 b	3.73 ± 0.33 a	3.39 ± 0.31 ab
Total root length	648.93 ± 72.70 a	743.72 ± 59.25 a	745.23 ± 55.46 a
Root diameter (mm)	0.23 ± 0.10 a	0.29 ± 0.04 a	0.30 ± 0.04 a
Root volume (mm^3^)	205.82 ± 116.00 a	240.91 ± 79.25 a	253.12 ± 62.50 a
Root surface area (mm^2^)	474.62 ± 261.27 a	676.10 ± 110.01 a	697.62 ± 95.89 a
Root projected area (mm^2^)	151.08 ± 83.17 a	215.21 ± 35.02 a	222.06 ± 30.53 a
Root tip number	398.25 ± 80.74 a	462.80 ± 40.42 a	471.80 ± 61.35 a

**Table 2:** General genomic features of KP3P9 and K13C.

Feature	KP3P9	K13C
	Chromosome	Chromosome
Genome topology	circular	circular
Assembly size (bp)	4,279,804	4,066,559
G + C content (%)	43.57	43.8
Protein coding genes	3.99	4,540
tRNA genes	97	86
rRNA genes	30	27
Prophage	4	4
Gene islands	6	6
CRISPR	4	5
Biosynthetic gene clusters	14	14
GenBank accession ID	JBQIBB000000000	JBQIBC000000000

**Table 3:** Predicted secondary metabolite biosynthetic gene clusters in KP3P9 and K13C genomes.

Strain Name	Cluster	Length/bp	Known Cluster	Species with Known Clusters	Similarity (%)
KP3P9	Cluster1	22,953	Sporulation killing factor	*Bacillus subtilis* subsp. *subtilis str.* 168	100
	Cluster2	65,391	Surfactin	*Bacillus velezensis* FZB42	82
	Cluster3	20,803	-	-	-
	Cluster4	114,758	Bacillaene	*Bacillus velezensis* FZB42	100
	Cluster5	77,759	Fengycin	*Bacillus velezensis* FZB42	100
	Cluster6	21,898	-	-	-
	Cluster7	20,170	Sublancin 168	*Bacillus subtilis* subsp. *subtilis str.* 168	100
	Cluster8	41,097	1-carbapen-2-em-3-carboxylic acid	*Pectobacterium carotovorum*	16
	Cluster9	51,777	Bacillibactin	*Bacillus subtilis* subsp. *subtilis str.* 168	100
	Cluster10	20,746	Pulcherriminic acid	*Bacillus subtilis* subsp. *subtilis str.* 168	100
	Cluster11	21,611	Subtilosin A	*Bacillus subtilis* subsp. *spizizenii* ATCC 6633	100
	Cluster12	41,418	Bacilysin	*Bacillus velezensis* FZB42	100
	Cluster13	20,269	-	-	-
	Cluster14	21,698	Thailanstatin A	*Burkholderia thailandensis*	10
K13C	Cluster1	65,391	Surfactin	*Bacillus velezensis FZB42*	82
	Cluster2	20,803	-	-	-
	Cluster3	26,295	-	-	-
	Cluster4	114,749	Bacillaene	*Bacillus velezensis FZB42*	100
	Cluster5	77,762	Fengycin	*Bacillus velezensis FZB42*	100
	Cluster6	21,898	-	-	-
	Cluster7	41,097	1-carbapen-2-em-3-carboxylic acid	*Pectobacterium carotovorum*	16
	Cluster8	51,777	Bacillibactin	*Bacillus subtilis* subsp. *subtilis str.* 168	100
	Cluster9	26,225	Subtilin	*Bacillus subtilis* subsp. *spizizenii* ATCC 6633	100
	Cluster10	20,746	Pulcherriminic acid	*Bacillus subtilis* subsp. *subtilis str.* 168	100
	Cluster11	21,611	Subtilosin A	*Bacillus subtilis* subsp. *spizizenii* ATCC 6633	100
	Cluster12	41,418	Bacilysin	*Bacillus velezensis* FZB42	100
	Cluster13	12,731	-	-	-
	Cluster14	21,698	Thailanstatin A	*Burkholderia thailandensis*	10

## Data Availability

The data presented in this study are openly available in GenBank, at https://www.ncbi.nlm.nih.gov/genbank/, reference number PV759774, PV759773, JBQIBB000000000 and JBQIBC000000000.

## Supplementary Materials

The following supporting information can be downloaded at https://www.mdpi.com/article/10.3390/microorganisms13102255/s1, Supplemental Figure S1: The circular maps of the KP3P9 (A) and K13C (B) genomes. Different colors represent different functional classifications of CDSs.

## Abbreviations

The following abbreviations are used in this manuscript:

BGC	Biosynthetic gene clusters
CRISPR	Clustered regularly interspaced short palindromic repeats
DSI	Disease severity index
*Foc*1	*F*. *oxysporum* f. sp. *cubense* race 1
*Foc*TR4	*F*. *oxysporum* f. sp. *cubense* tropical race 4
*Fs*	*F. solani*
IAA	Indole-3-acetic acid
LB	Lysogeny broth
MSR	Maize stalk rot
PDA	Potato dextrose agar
PGP	Plant-growth-promoting
Rs	*Rhizoctonia solani*
